# Tear Proteome Analysis with Patients with Primary Nasolacrimal Duct Obstruction

**DOI:** 10.3390/ijms26136449

**Published:** 2025-07-04

**Authors:** Wonseok Bang, Heejeong You, Jong-Moon Park, Junyoung Park, Byeongsoo Kang, Minjung Ju, Yelin Park, Hookeun Lee, Seunghoon Back, Helen Lew

**Affiliations:** 1 Basil Biotech, Incheon 21984, Republic of Korea; lebenlied@gachon.ac.kr (W.B.); basil@basilbiotech.com (J.-M.P.); jypark@basilbiotech.com (J.P.); bskang@basilbiotech.com (B.K.); mjjoo@basilbiotech.com (M.J.); apfjd8520@gmail.com (Y.P.); 2 College of Pharmacy, Gachon University, Incheon 21936, Republic of Korea; hklee@gachon.ac.kr; 3 Department of Ophthalmology, CHA Bundang Medical Center, CHA University, Seongnam-si 13496, Republic of Korea; hjyoueye@gmail.com; 4 Department of Chemistry and Center for Proteogenome Research, Korea University, Seoul 02843, Republic of Korea

**Keywords:** nasolacrimal duct obstruction, epiphora, tear proteomics, LC-MS/MS, biomarkers

## Abstract

The pathogenesis of primary acquired nasolacrimal duct obstruction (PANDO) remains unclear, with several factors implicated, including anatomical structures, hormones, and tear components. This study explored tear proteins to better understand PANDO etiology by comparing protein expression in tears from patients with PANDO and healthy controls. Tear samples were collected from 22 patients with PANDO (mucous and membranous types) and 8 controls using Weck-Cel sponges. Protein analysis was conducted using LC-MS/MS to identify and quantify tear proteins. Female patients with PANDO had higher numbers of differentially expressed proteins (DEPs) compared with males. Certain DEPs associated with inflammatory pathways or the lacrimal duct epithelium, including SERPINB1, SERPINA3, CTSG, SLPI, and EZR, were identified in male patients. Although this is a preliminary study, our results offer insights into the pathogenesis of PANDO, with potential to distinguish between mucous and membranous subtypes. The potential biomarkers identified in this study could enhance early diagnosis and treatment, shedding light on inflammatory and immune processes in PANDO.

## 1. Introduction

Epiphora is caused by disruption of the balance of tear production, evaporation, absorption, and drainage. Primary acquired nasolacrimal duct obstruction (PANDO) is a common ophthalmic disorder causing epiphora in adults, mainly interfering with tear absorption and drainage. Based on dacryoendoscopic findings, the obstruction pattern of PANDO can be divided into membranous, mucinous, and dacryolith types [1].

Previous studies have proposed multiple etiologies of PANDO [2]. Structural factors include anatomical variation in the nasolacrimal duct, the vascular structure of the lacrimal system, and the morphology of the nose and paranasal sinuses. Functional factors of the lacrimal drainage system include hormonal regulation, tear components, surfactants, antimicrobial defense mechanisms, and lacrimal drainage-associated lymphoid tissue. Additionally, the system hosts a diverse microbial community, known as the lacriome, which interacts with these factors to support ocular immunity and overall eye health.

A deeper understanding of tear composition could shed light on the pathogenesis of nasolacrimal duct obstruction. Research teams have explored various tear parameters, including meniscus height, osmolarity, pH, and electrolyte balance, to explore their potential role in this condition [3,4,5]. Recent research has identified tear components, particularly proteins, as potential contributors to nasolacrimal duct obstruction. Several studies have specifically examined inflammatory cytokines in tear fluid, reporting elevated levels of certain cytokines in the tears of patients with PANDO [6,7,8]. Additionally, one study reported a decrease in the levels of proteins, albumin, and lactoferrin compared with healthy controls [9]. The same study noted the normalization of decreased protein levels after surgical treatment by nasolacrimal duct intubation and following extubation.

Recent advances in mass spectrometry have enabled the analysis of tear proteomics in various ocular diseases [10,11]. Liquid chromatography–mass spectrometry (LC-MS) facilitates the identification and quantification of proteins, revealing differentially expressed proteins (DEPs) across conditions. DEPs are determined according to differences in the relative abundance of the same protein between groups, indicating changes in protein regulation. Previous studies have used MS to analyze the tear proteome profile in various ocular diseases, including dry eye disease (DED), meibomian gland dysfunction, Sjögren’s syndrome, thyroid-associated ophthalmopathy, glaucoma, and keratoconus [12,13]. Variations in protein expression levels have enabled differentiation not only between disease and normal states but also among distinct disease groups, making it possible, for example, to distinguish meibomian gland dysfunction (MGD) from DED based on the differential expression of specific proteins [14].

In this study, we assessed the tear protein profiles of patients with PANDO based on obstruction type. We also analyzed DEPs across subgroups, identifying specific proteins potentially linked to obstruction.

## 2. Results

### 2.1. Clinical Characteristics of the Study

This study included 30 patients: 22 diagnosed with PANDO and 8 controls (Table 1). The groups did not differ significantly in terms of age or sex. Among the 22 patients with obstruction, dacryoendoscopic findings classified 10 as having the membranous type and 12 as having the mucinous type. The Mann–Whitney U and chi-square tests were used to compare the groups.

### 2.2. Comprehensive Global Tear Proteome

**Figure 1:**
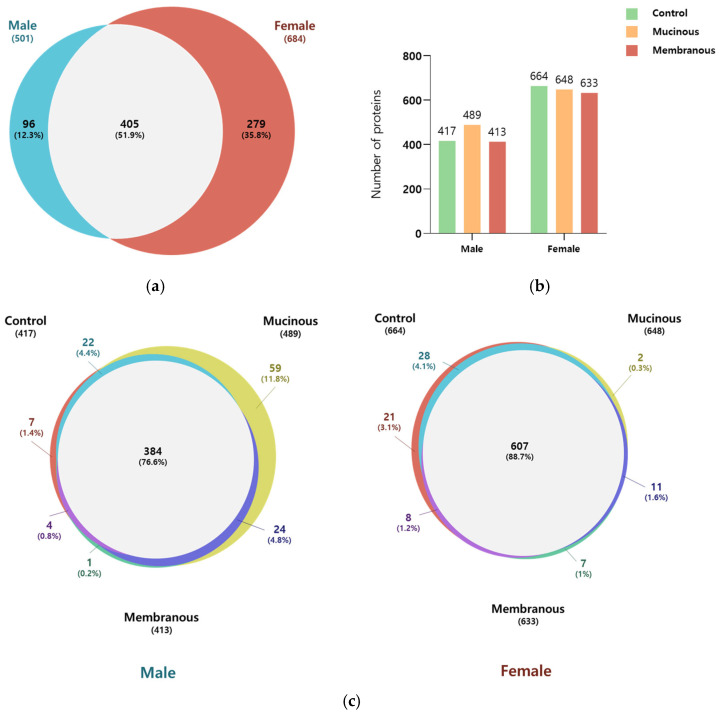
Comparison of identified proteins in male subjects and female subjects and across subgroups. (**a**) Venn diagram illustrating the overlap in the number of identified proteins in males and females. (**b**) Bar graph showing the number of proteins in the control, mucinous, and membranous groups for males and females. (**c**) Venn diagram representing the number of overlapping proteins among the control, mucinous, and membranous groups in males and females.

In total, 780 proteins were identified across all tear samples, with a false discovery rate (FDR) of 1% at the protein and peptide–spectrum match (PSM) level (Figure 1; Table S1). Among these, 501 and 684 were detected in male (*n* = 13) and female (*n* = 17) subjects, respectively. Of the proteins, 51.9% (405 proteins) were common to both sexes. The Venn diagrams in Figure 1 illustrate the distribution of proteins for each sex according to obstruction type. In male subjects, 76.6% of identified proteins were found in all three obstruction subgroups, whereas in female subjects, 88.7% of proteins were shared across all three subgroups.

### 2.3. Differentially Expressed Proteins in Patients with PANDO Compared to the Control Group and Across Subgroups in Males and Females

**Figure 2:**
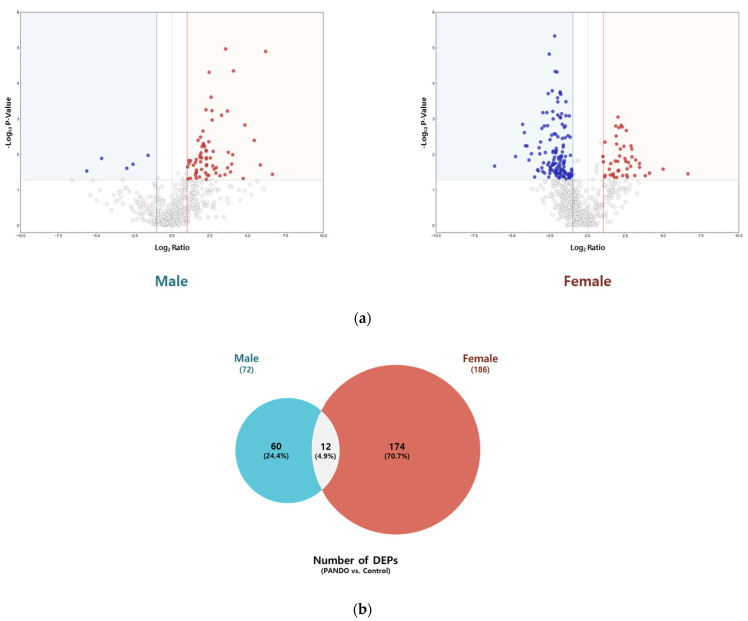

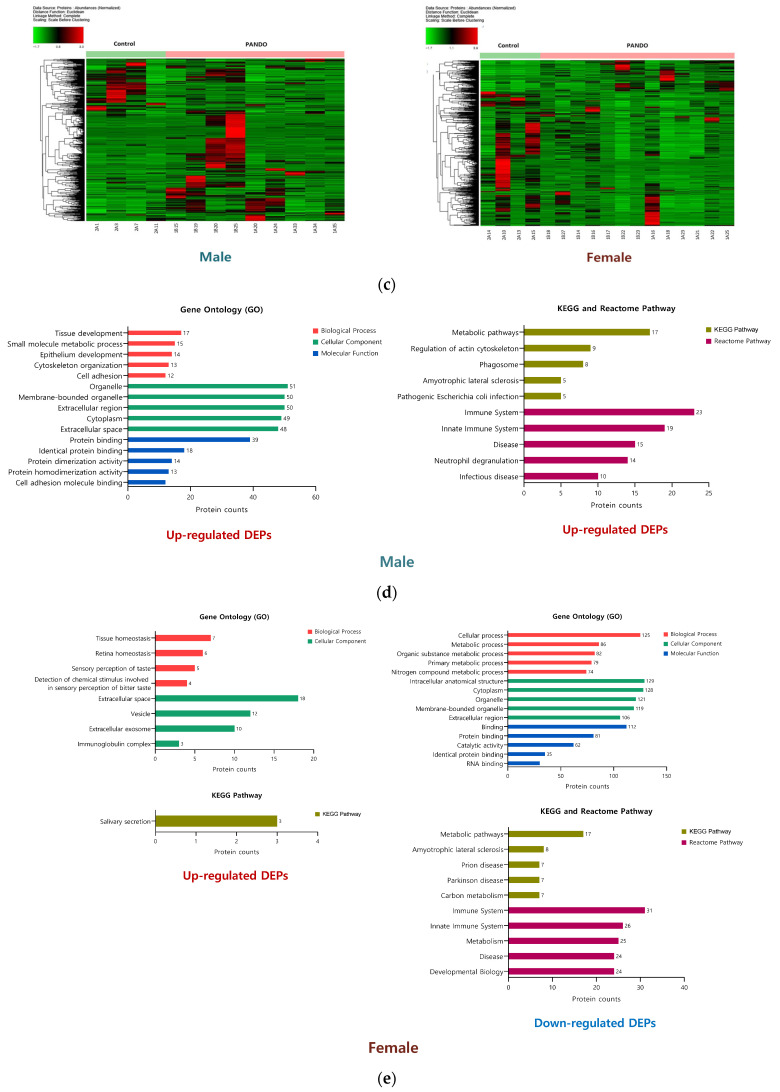
Tear proteome analysis between PANDO and control groups in males and females. (**a**) Volcano plot for comparison of significantly differentially expressed proteins (DEPs) in males and females. Red spots represent upregulated proteins with a threshold of Log_2_Ratio ≥ 1 and *p*-Value ≤ 0.05. Blue spots indicate downregulated proteins with a threshold of Log_2_Ratio ≤ −1 and *p*-Value ≤ 0.05. (**b**) Venn diagram illustrating the overlap in the number of DEPs in males and females. (**c**) Hierarchical clustering of the normalized abundances of commonly identified proteins in the control and PANDO groups in males and females. (**d**) Gene Ontology (GO) enrichment, KEGG pathway, and Reactome pathway of upregulated proteins in males. (**e**) Gene Ontology (GO) enrichment, KEGG pathway, and Reactome pathway of upregulated and downregulated proteins in females.

We identified 72 and 186 DEPs in patients with PANDO compared to controls in males (control, *n* = 4; PANDO, *n* = 9) and females (control, *n* = 4; PANDO, *n* = 13), respectively (Figure 2; Table S2). Among these, 12 overlapped between the sexes, including IGHV3–66, IGHV3–30, CD59, MUC16, IGKV3–20, SBSN, CRABP2, PKM, TYMP, H4C1, F5H423 (TGc domain-containing protein), and A0A494C0J7 (uncharacterized protein). In subgroup analyses, among males, 76 DEPs were unique to the mucus group while 27 were unique to the membranous group. Similarly, in females, 123 were unique to the mucus group whereas 161 were unique to the membranous group. Next, a comprehensive STRING network analysis was conducted on significant DEPs (Log2Ratio ≥ |2|, *p*-Value ≤ 0.05) observed in patients with PANDO versus controls and within subgroups. This included Gene Ontology (GO) enrichment, KEGG pathway, and Reactome pathway analyses to explore their functional and biological significance (Table S3). Notably, in males, DEPs were associated with neutrophil degranulation- and immune system-related pathways, indicating the enrichment of innate immune-related processes.

### 2.4. Selection of Candidate Biomarkers for Type of Nasolacrimal Duct Obstruction in Males

**Figure 3:**
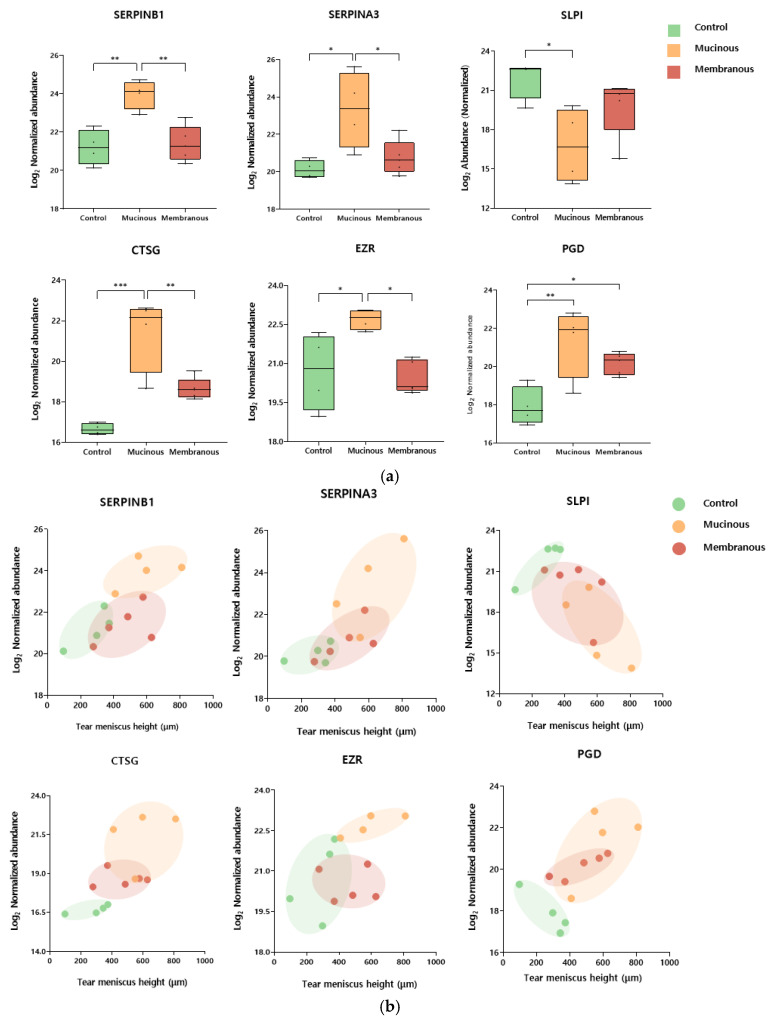
Tear meniscus height (TMH)-associated candidate biomarkers for type of nasolacrimal duct obstruction in males. (**a**) Box plots showing the normalized abundance of leukocyte elastase inhibitor (SERPINB1), alpha-1-antichymotrypsin (SERPINA3), antileukoproteinase (SLPI), cathepsin G (CTSG), ezrin (EZR), and 6-phosphogluconate dehydrogenase (PGD) as candidate biomarkers for distinguishing between the control, mucinous, and membranous groups in males. * *p*-Value ≤ 0.05, ** *p*-Value ≤ 0.01, and *** *p*-Value ≤ 0.001. (**b**) Scatter plots showing the relationship between tear meniscus height (TMH) and the normalized abundance of the candidate biomarkers. Each point represents an individual sample, color-coded by group: control (green), mucinous (orange), and membranous (red). Ellipses were manually drawn to visually indicate group-specific clustering patterns.

Several biomarker candidates were selected among DEPs based on statistical significance and Gene Ontology, KEGG pathway, and Reactome pathway analyses. Notably, among males, selected proteins included leukocyte elastase inhibitor (SERPINB1), cathepsin G (CTSG), 6-phosphogluconate dehydrogenase (PGD), alpha-1-antichymotrypsin (SERPINA3), ezrin (EZR), cathepsin D (CTSD), coronin-1A (CORO1A), antileukoproteinase (SLPI), lactoperoxidase (LPO), glucose-6-phosphate isomerase (GPI), elongation factor 1-delta (EEF1D), annexin A4 (ANXA4), mucin-16 (MUC16), isoform 2 of proline-rich protein 4 (PRR4), cathepsin B (CTSB), alpha-1-acid glycoprotein 2 (ORM2), metalloproteinase inhibitor 1 (TIMP1), leukotriene A-4 hydrolase (LTA4H), and alpha-2-hs-glycoprotein (AHSG). Table 2 presents the results of analyses of the relationship between tear meniscus height and the levels of selected biomarkers. SERPINB1, SERPINA3, CTSG, SLPI, EZR, and PGD were statistically and significantly correlated with tear meniscus height. Figure 3 illustrates the expression levels of these proteins across obstruction types and their correlations with tear meniscus height.

## 3. Discussion

PANDO has a multifactorial etiology involving anatomical, hormonal, immune, and environmental influences, as well as disruptions in antimicrobial defenses. Analyses of tear proteomics revealed sex-specific patterns as key findings, with substantial differences in tear protein profiles and DEPs in patients with PANDO compared with controls.

LTF, SCGB2A1, haptoglobin, α1-antitrypsin (SERPINA1), CST4, LCN1, and LACRT are reportedly upregulated in the tear fluid of female patients compared with males [10]. Additionally, LC-MS/MS analysis has revealed significant upregulation of tear proteins in healthy female samples compared with male samples [15].

To address this sex-based disparity in protein expression, we stratified and analyzed data separately by sex, with the goal of identifying sex-specific biomarkers and mechanisms relevant to each group.

Only 12 DEPs were shared between males and females, a markedly small number given that 72 DEPs were identified in males and 186 in females. Notably, the number of DEPs in females was more than twice that observed in males, suggesting distinct protein expression patterns between sexes and implying greater protein diversity associated with obstruction in females. This aligns with previous reports of a higher prevalence of PANDO among women, potentially linked to hormonal and anatomical differences [16,17,18]. These findings underscore the importance of sex-specific analyses in investigating the pathophysiology of nasolacrimal duct obstruction.

To translate these findings into clinical practice, it is crucial to clarify the correlation between DEPs and the clinical symptom of epiphora. Therefore, after analyzing DEPs by sex and identifying related pathways through STRING analysis, we examined the abundance of each protein and possible correlations with tear meniscus height [19].

In females, neutrophil collagenase (matrix metalloproteinase-8, MMP8), lipocalin-1 (LCN1), galectin-3-binding protein (LGALS3BP), and monocyte differentiation antigen CD14 (CD14) were significantly correlated with tear meniscus height (Appendix A). MMP-8, primarily secreted by neutrophils, helps degrade collagen in connective tissues. It is also associated with inflammatory processes and ocular diseases, including atopic conjunctivitis and ocular rosacea [20,21].

In male patients, tear meniscus height was associated with SERPINB1, SERPINA3, CTSG, EZR, SLPI, and PGD. Notably, the first four were differentially expressed between mucous and membranous PANDO subtypes, making them potentially useful biomarkers for subtype analysis.

Cathepsin G is a serine protease primarily found in neutrophils and is associated with antimicrobial defense, inflammation, and tissue remodeling [22]. Under normal conditions, it acts as a key component against infection, killing pathogens and activating the inflammatory response. However, excessive activity can cause tissue damage and chronic inflammation. Several protease inhibitors, including SERPINA3, SERPINB1, and SLPI, can regulate its activity.

SERPINA3 has also been reported in association with multiple ocular diseases, including anterior uveitis and Graves’ orbitopathy [23,24,25]. SERPINB1 is mainly found in neutrophils and lung epithelia, protecting tissues by inhibiting cathepsin, neutrophil elastase, and proteinase 3 [26]. By regulating the activity of elastases, it prevents excessive degradation of extracellular matrix (ECM) components, such as collagen and elastin. Finally, SLPI, secreted by epithelial cells and found in mucosal fluids, not only inhibits cathepsin G but also has antimicrobial and anti-inflammatory properties, protecting mucosal surfaces from excessive protease activity and infections [27]. Together, these three inhibitors maintain a critical balance, effectively regulating the function of cathepsin G without causing excessive inflammation or tissue destruction.

The overexpression of cathepsin G and underexpression of SERPINB1 and SLPI have been reported in muco-obstructive respiratory diseases such as cystic fibrosis and COPD [28,29,30]. The overexpression of cathepsin G leads to ECM degradation and increased mucus secretion in affected tissues. A similar pathogenesis may be involved in PANDO, as the nasolacrimal duct consists of columnar epithelial cells and goblet cells, similar to the respiratory tract. Our findings revealed excessive overexpression of cathepsin G in patients with PANDO (20-fold increase) and downregulation of SLPI (6-fold decrease), consistent with previous reports. Chronic inflammation in nasolacrimal duct obstruction may result from an imbalance between neutrophil elastase and its inhibitors, leading to ECM degradation, mucus production, and the progressive narrowing of the duct. Thus, proteins such as SERPINB1 and cathepsin G may serve as both biomarkers of inflammation and potential therapeutic targets for mitigating tissue damage and the progression of obstruction.

The upregulation of PGD was observed in patients with PANDO, regardless of the obstruction type. PGD is an enzyme involved in producing NADPH, which is essential for antioxidant defense mechanisms [31]. Although PGD has been reported to be upregulated in various cancers, its role in tear proteomics is poorly understood [32]. A previous study reported the upregulation of PGD in patients with keratoconus, but the underlying mechanisms have not been fully elucidated [33]. We cautiously propose that the increased oxidative burden associated with inflammation in patients with PANDO may account for the observed PGD upregulation.

The differentiation of PANDO into mucous and membranous subtypes based on dacryoendoscopic findings enabled deeper exploration of subtype-specific pathogenesis. Notably, SERPINB1, SERPINA3, CTSG, and EZR emerged as novel markers for mucous obstruction. These proteins are linked to inflammation, cellular cytoskeletal organization, and antimicrobial activity, suggesting that mucous obstruction may involve an inflammatory response originating from the lacrimal drainage system.

The involvement of ezrin in the mucous obstruction of PANDO suggests a role in epithelial remodeling and inflammation-related changes in the lacrimal drainage surface structure. The nasolacrimal duct epithelium consists of ciliated and non-ciliated cells with microvilli [34,35]. In the development and maintenance of microvilli, ezrin functions as a key structural and regulatory protein that links the plasma membrane to the actin cytoskeleton [36,37].

Also, ezrin is an important linker protein that binds with transmembrane mucins, such as MUC16. Although not much is known about the expression of ezrin in the nasolacrimal duct, previous studies have demonstrated that the EZR–MUC16 interaction is essential for maintaining the integrity of the ocular surface and preventing pathogen adhesion [38,39]. Although further studies are required, it is plausible that increased ezrin expression could reflect a remodeling process in response to inflammation or obstruction to maintain the lacrimal duct surface.

Targeting neutrophil elastase activity using inhibitors or anti-inflammatory therapies may help preserve ECM integrity and prevent further ductal narrowing. Therapeutic strategies aimed at enhancing epithelial repair and stabilizing the cytoskeletal architecture could mitigate the effects of inflammation and promote functional recovery. These findings not only provide mechanistic insights into the pathogenesis of mucous-type PANDO but also open new avenues for subtype-specific diagnostics and therapies.

The identification of these biomarkers establishes a foundation for developing diagnostic tools and targeted therapies. For example, tear proteomics could be leveraged to identify specific DEPs in clinical settings, enabling early diagnosis of PANDO and its subtypes without requiring invasive procedures.

With regard to sex-based differences in protein expression, we stratified and analyzed the data separately by sex. We prioritized common proteins across both sexes to identify universally expressed markers while highlighting distinct proteins as secondary findings. Future research should focus on collecting hormonal profiles (e.g., estrogen, progesterone, testosterone) from patients during sampling to assess correlations with protein expression data. Further analysis of protein expression in relation to the hormonal cycle in females (e.g., pre- or post-menopausal status) and increasing the sample size could help clarify sex-specific variation. Additionally, further pathway enrichment analyses (e.g., Gene Ontology, KEGG pathway, and Reactome pathway) would be useful to determine whether male- and female-specific proteins are linked with similar biological processes.

Because tears flow along the mucosal layer of the lacrimal drainage system, changes in tear components may influence the lacrimal mucosa and contribute to nasolacrimal duct obstruction. Conversely, changes in tear composition could result from obstruction, reflecting changes in the lacrimal pathway. To further investigate this relationship, analyses of fluid collected from the lacrimal duct may provide valuable insights.

Despite its strengths, this study had certain limitations. This study was a relatively small, exploratory study conducted to discover DEPs in patients with PANDO. The small sample size in each group may have reduced the statistical power and compromised the reliability of some statistically significant findings. Furthermore, the limited sample size may restrict the generalizability of the findings to a broader population. Future studies employing larger cohorts are needed. Also, the protein analysis in this study was performed using the LC-MS/MS technique. Additional complementary techniques to provide orthogonal validation would further solidify the results. Additionally, tear samples were collected using noninvasive methods; while clinically applicable, these techniques may not have fully captured all protein variations present in the lacrimal drainage system.

## 4. Materials and Methods

### 4.1. Subjects

Patients were included in this study if they were diagnosed with PANDO and underwent dacryoendoscopy-assisted nasolacrimal duct silicone tube intubation at Bundang CHA Hospital between August and December 2023. PANDO was diagnosed in patients who presented with epiphora and had a Munk score ≥ 2, reflux during syringing, and dacryocystographic evidence of obstruction, including prominent lacrimal duct narrowing, complete obstruction, and secondary dilation. Among patients with unilateral PANDO, the contralateral healthy eye was included as the control group. None of the control eyes exhibited signs of epiphora, and all had normal syringing results and normal dacryocystographic findings. Obstruction was classified into membranous and mucous subtypes based on dacryoendoscopic findings, as previously reported [1].

### 4.2. Tear Sample Collection

**Figure 4:**
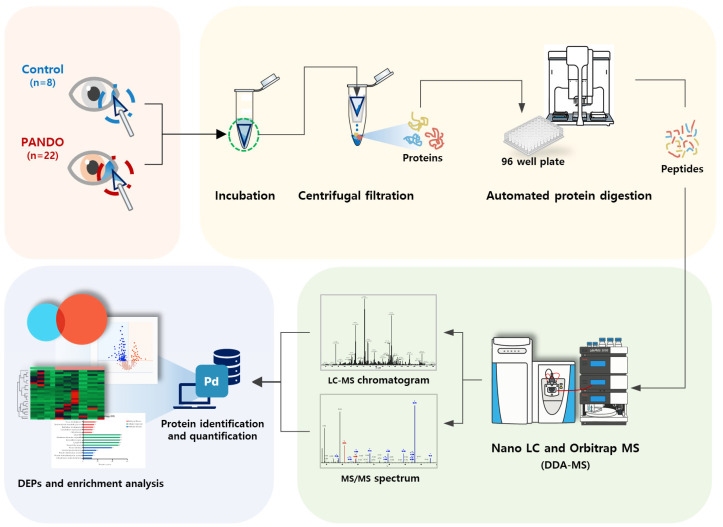
Schematic diagram of proteomic experiments.

Tear samples were collected from the lower conjunctival sac using a Weck-Cel ophthalmic sponge (BVI Medical, Waltham, MA, USA). To prevent potential changes in tear composition, topical anesthesia was not administered. Only the sponge tip was placed in a conical tube and stored at −80 °C until protein analysis. The entire experimental procedure followed the workflow illustrated in Figure 4.

### 4.3. Protein Analysis

#### 4.3.1. Chemicals and Reagents

High-performance liquid chromatography (HPLC)-grade water, acetonitrile, and methanol were purchased from JT Baker (Philipsburg, NJ, USA). Urea, iodoacetamide, ammonium bicarbonate, trifluoroacetic acid, and formic acid were purchased from Sigma-Aldrich (St. Louis, MO, USA). Tris(2-carboxyethyl) phosphine (TCEP) was purchased from Thermo Fisher Scientific (Rockford, IL, USA). Sequencing-grade modified trypsin was purchased from Promega (Madison, WI, USA).

#### 4.3.2. Protein Digestion

The sponge was lysed using lysis buffer (8 M urea–0.1 M Tris-HCl, pH 8.5), and proteins were eluted with an Amicon^®^ Ultra Centrifugal Filter (100 kDa). Protein concentration was quantified using the Pierce BCA Protein Assay Kit (Thermo Fisher Scientific, Waltham, MA, USA), after which 45 µg of protein from each sample was lyophilized for automated filter-aided sample preparation (aFASP). Protein digestion was carried out on a liquid-handling robotic system (Agilent Technologies, Santa Clara, CA, USA) integrated with an automated platform controlled by VWorks software (11.4.0.1233), following a previously optimized protocol [40]. Briefly, samples were reduced with 5 mM tris(2-carboxyethyl) phosphine for 30 min at 33 °C on a 96-well filter plate, followed by alkylation with 50 mM iodoacetamide for 1 h at 25 °C in the dark. After sequential washing with lysis buffer and 50 mM ammonium bicarbonate, proteins were digested with trypsin at an enzyme-to-protein ratio of 1:50 (*w*/*w*) at 37 °C for 18 h. The resulting peptides were eluted, dried using a SpeedVac (Gyrozen Co., Ltd., Seoul, Republic of Korea), and dissolved in 0.1% trifluoroacetic acid in water. Next, they were desalted using a C18 spin column, dried again, and stored at −20 °C until LC-MS/MS analysis.

#### 4.3.3. LC-MS/MS Analysis

The prepared samples were resuspended in 0.1% formic acid in water and analyzed using a Q-Exactive Orbitrap hybrid mass spectrometer (Thermo Fisher Scientific, Waltham, MA, USA) coupled with an Ultimate 3000 system (Thermo Fisher Scientific). Each sample was injected at 500 ng for analysis. A 2 cm × 75 μm ID trap column packed with 3 μm C18 resin and a 50 cm × 75 μm ID analytical column packed with 2 μm C18 resin were used to separate peptides based on hydrophobicity. The mobile phase solvents consisted of 0.1% formic acid in water (A) and 0.1% formic acid in 80% acetonitrile (B), with a fixed flow rate of 300 nL/min. The gradient of the mobile phase was as follows: 4% solvent B for 14 min, 4–15% solvent B for 61 min, 15–28% solvent B for 50 min, 28–40% solvent B for 20 min, 40–96% solvent B for 2 min, holding at 96% of solvent B for 13 min, 96–4% solvent B for 1 min, and 4% solvent B for 24 min. A data-dependent acquisition method was employed, selecting the top 10 precursor peaks for isolation and fragmentation. Ions were scanned at high resolution, set at 70,000 in MS1 and 17,500 in MS2 at *m*/*z* 400. The MS scan range covered 400–2000 m/z at both MS1 and MS2 levels. Precursor ions were fragmented using a normalized collisional energy (NCE) of 27%, and dynamic exclusion was set to 30 s. LC-MS chromatograms are provided in Figure S1.

#### 4.3.4. Data Analysis and Statistics

MS/MS raw files from each analysis were processed using Proteome Discoverer™ software (ver. 3.1), with the Homo sapiens database downloaded from Uniprot (2022.05). The consensus workflow incorporated peptide–spectrum match (PSM) validation and SEQUEST HT as the database search algorithm. Search parameters were set to a precursor ion mass tolerance of 10 ppm, a fragment ion mass tolerance of 0.02 Da, and a maximum of two missed cleavages with trypsin. Dynamic modifications included static carbamidomethylation of cysteine (+57.021 Da), variable methionine oxidation (+15.995 Da), acetylation of the protein N-terminus (+42.011 Da), and carbamylation of the protein N-terminus (+43.006 Da). Results below a 1% FDR were selected and filtered for peptides of at least six residues in length. Normalization was performed using the total peptide abundance, and missing values were imputed using the low-abundance resampling method, which replaces them with random values sampled between the minimum and the 5th percentile of all detected values. Fold change was calculated based on protein abundance ratios, and *p*-values for quantification ratios were computed using a *t*-test against the background. DEPs were subjected to enrichment analyses of GO biological processes, cellular components, and molecular functions, as well as KEGG pathways [41] and Reactome pathways, using STRING version 12.0.

## 5. Conclusions

We conducted a comprehensive tear proteomic analysis of patients with PANDO and observed significant sex-specific and subtype-specific differences in protein expression. Distinct DEPs were identified in males and females, highlighting the importance of considering sex in proteomic research. Proteins including cathepsin G, leukocyte elastase inhibitor, alpha-1-antichymotrypsin, and ezrin emerged as exploratory biomarkers, particularly in mucous-type obstructions, offering insights into the inflammatory pathogenesis underlying PANDO. Further investigations into tear proteomics will deepen the understanding of disease mechanisms and facilitate earlier, noninvasive diagnosis of PANDO.

## Figures and Tables

**Table 1:** Patient demographics.

Characteristics	PANDO	Control	*p*-Value
Number of patients	22 (73.3%)	8 (26.7%)	-
Membranous	10	-	-
Mucinous	12	-	-
Age	64.86 ± 10.99	61.88 ± 14.15	0.629
Gender (M:F)	9:13	4:4	0.657
Side (OD:OS)	12:10	5:3	0.697
Tear meniscus height (μm)	470.59 ± 186.00	278.63 ± 159.15	0.015

**Table 2:** Correlation analysis of protein abundancy with tear meniscus height (μm) in male patients.

			Tear Meniscus Height	
Protein (Gene Name)	Average Normalized Abundances		Correlation Coefficient	*p*-Value
	PANDO	Control		
Leukocyte elastase inhibitor (*SERPINB1*)	9.75 × 10^6^	2.79 × 10^6^	0.626	0.007
Cathepsin G (*CTSG*)	2.07 × 10^6^	1.05 × 10^5^	0.595	0.01
6-phosphogluconate dehydrogenase (*PGD*)	2.42 × 10^6^	2.96 × 10^5^	0.565	0.015
Alpha-1-antichymotrypsin (*SERPINA3*)	9.94 × 10^6^	1.19 × 10^6^	0.565	0.015
Ezrin (*EZR*)	4.01 × 10^6^	2.38 × 10^6^	0.504	0.03
Cathepsin D (*CTSD*)	2.84 × 10^6^	6.66 × 10^5^	0.504	0.03
Coronin-1A (*CORO1A*)	2.74 × 10^6^	7.37 × 10^5^	0.473	0.042
Antileukoproteinase (*SLPI*)	9.97 × 10^5^	5.22 × 10^6^	−0.473	0.042
Lactoperoxidase (*LPO*)	1.43 × 10^6^	1.92 × 10^6^	−0.443	0.057
Glucose-6-phosphate isomerase (*GPI*)	2.04 × 10^6^	2.92 × 10^5^	0.443	0.057
Elongation factor 1-delta (*EEF1D*)	4.73 × 10^5^	3.77 × 10^4^	0.412	0.076
Annexin A4 (*ANXA4*)	9.84 × 10^4^	2.22 × 10^4^	0.382	0.1
Mucin-16 (*MUC16*)	2.84 × 10^5^	3.23 × 10^4^	0.382	0.1
Isoform 2 of proline-rich protein 4 (*PRR4*)	4.43 × 10^5^	1.51 × 10^4^	0.351	0.131
Cathepsin B (*CTSB*)	1.87 × 10^6^	1.77 × 10^6^	−0.321	0.168
Alpha-1-acid glycoprotein 2 (*ORM2*)	3.26 × 10^4^	3.76 × 10^5^	−0.29	0.212
Metalloproteinase inhibitor 1 (*TIMP1*)	9.25 × 10^5^	2.86 × 10^5^	0.198	0.393
Leukotriene A-4 hydrolase (*LTA4H*)	1.23 × 10^5^	3.02 × 10^4^	0.198	0.393
Alpha-2-HS-glycoprotein (*AHSG*)	2.58 × 10^4^	9.39 × 10^5^	−0.198	0.393

## Data Availability

The mass spectrometry proteomic data are available on the ProteomeXchange Consortium via the PRIDE partner repository with the dataset identifier PXD063414.

## Supplementary Materials

The following supporting information can be downloaded at https://www.mdpi.com/article/10.3390/ijms26136449/s1.

## Abbreviations

The following abbreviations are used in this manuscript:

PANDO	Primary acquired nasolacrimal duct obstruction
DEPs	Differentially expressed proteins
ECM	Extracellular matrix

## Appendix A

**Table A1:** Correlation analysis of protein abundancy with tear meniscus height in female patients.

			Tear Meniscus Height	
Protein (Gene Name)	Average Normalized Abundances		Correlation Coefficient	*p*-Value
	PANDO	Control		
Neutrophil collagenase (*MMP8*)	1.15 × 10^5^	2.13 × 10^4^	0.485	0.007
Lipocalin-1 (*LCN1*)	8.71 × 10^9^	2.46 × 10^9^	0.368	0.039
Galectin-3-binding protein (*LGALS3BP*)	1.43 × 10^7^	4.21 × 10^6^	0.368	0.039
Monocyte differentiation antigen CD14 (*CD14*)	6.29 × 10^5^	2.05 × 10^5^	0.368	0.039
Lactoferrin (*LTF*)	3.52 × 10^9^	9.13 × 10^8^	0.309	0.084
Keratin, type 1 cytoskeletal 14 (*KRT14*)	1.33 × 10^8^	8.11 × 10^8^	−0.309	0.084
Plakophilin-1 (*PKP1*)	1.79 × 10^6^	7.71 × 10^6^	−0.294	0.099
Azurocidin (*AZU1*)	5.68 × 10^5^	5.14 × 10^4^	0.294	0.099
Zinc-alpha-2-glycoprotein (*AZGP1*)	3.92 × 10^8^	1.56 × 10^8^	0.265	0.138
Desmoglein-1 (*DSG1*)	1.45 × 10^7^	6.53 × 10^7^	−0.235	0.187
Annexin (*ANXA2*)	1.50 × 10^7^	4.80 × 10^7^	−0.221	0.217
Alpha-2-macroglobulin-like protein (*A2ML1*)	1.35 × 10^6^	6.31 × 10^6^	−0.221	0.217
Voltage-dependent anion-selective channel protein 1 (*VDAC1*)	3.51 × 10^5^	2.53 × 10^6^	−0.221	0.217
Deleted in malignant brain tumors 1 protein (*DMBT1*)	1.66 × 10^8^	2.41 × 10^7^	0.206	0.249
Interleukin-36 gamma (*IL36G*)	2.75 × 10^5^	3.63 × 10^6^	−0.206	0.249
Lysozyme C (*LYZ*)	4.80 × 10^9^	1.73 × 10^9^	0.176	0.323
Cystatin-S (*CST4*)	1.95 × 10^8^	4.28 × 10^7^	0.176	0.323
Prolactin-inducible protein (*PIP*)	4.75 × 10^8^	1.11 × 10^8^	0.162	0.365
Desmocollin-1 (*DSC1*)	7.39 × 10^6^	2.32 × 10^7^	−0.147	0.410
LACRTF	2.98 × 10^6^	4.86 × 10^5^	0.147	0.410
Elongation factor 2 (*EEF2*)	3.32 × 10^6^	1.22 × 10^7^	−0.132	0.458
Extracellular glycoprotein lacritin (*LACRT*)	3.53 × 10^8^	6.70 × 10^7^	0.118	0.510
Calmodulin-like protein 5 (*CALML5*)	6.76 × 10^6^	1.24 × 10^8^	−0.088	0.621
Polymeric immunoglobulin receptor (*PIGR*)	9.18 × 10^8^	1.58 × 10^8^	0.059	0.742
Tubulin alpha-4A chain (*TUBA4A*)	6.69 × 10^5^	2.84 × 10^6^	−0.059	0.742
Zymogen granule protein 16 homolog B (*ZG16B*)	1.86 × 10^7^	7.14 × 10^6^	0.029	0.869
